# Supplementary material for: Extensive topographic remapping and functional sharpening in the adult rat visual pathway upon first visual experience
Source: PLoS Biol. 2023 Aug 17;21(8):e3002229. doi: 10.1371/journal.pbio.3002229 (PMC10434970; doi:10.1371/journal.pbio.3002229)
Supplement: Attachment — Submitted filename: Response to Editors.pdf [file pbio.3002229.s002.pdf]

Associate Principal Investigator
Champalimaud Research
Champalimaud Centre for the Unknown
Lisbon, 1400-038, Portugal
+351 210 380 000 ext. 4467
Noam.Shemesh@neuro.fchampalimaud.org
http://shemeshlab.org

Director, Champalimaud preclinical MRI Centre (CMC)

Champalimaud Foundation

Lisbon, Portugal 06/02/2023

Subject: Response Letter re submission (PBIOLOGY-D-23-00116).

Dear Dr. Dickson,

Thank you for assessing our initial submission and for your comment on our manuscript "*Extensive topographic remapping and functional sharpening in the adult rat visual pathway upon first visual experience*" (PBIOLOGY-D-23-00116).

We strongly believe that the issue raised on the exposure to red light is in fact a non-issue. The animals were "exposed" to red light for **less than two minutes once a week** during husbandry. The wavelength of the light source used for husbandry was 620nm, and the filter we used even further attenuated any residual spectral lines emitting below 580nm. Although the sensitivity of rats to red light has been recently debated, studies suggest that rat light sensitivity peaks at 362 nm and 502 nm for UV and M-cones, respectively, and that rats are insensitive for wavelengths above 620 nm (Deegan and Jacobs 1993; Rocha et al. 2016; Jacobs, Neitz, and Deegan 1991). Given the source and filters we used, and the very short duration of exposure, we maintain that the rats are well within conventional dark-rearing regimes, as is also corroborated by our experimental results.

We completely agree that this should have been more clearly stated in our manuscript and we apologize for this confusion. We have now edited the manuscript providing a comprehensive explanation of the visual deprivation paradigm (lines 510-526). We also now elaborate on this in the Discussion section (481-485).

Given these clarifications, we trust that you will agree that the animals saw perceptual light for the first time in the scanner, as prescribed.

We trust that the paper is now suitable for peer review and look forward to hearing from you on your decisions.

Thank you and Best Wishes,
Joana Carvalho and Noam Shemesh

References:

Deegan, J. F., 2nd, and G. H. Jacobs. 1993. "On the Identity of the Cone Types of the Rat Retina." Experimental Eye Research 56 (3): 375–77.
Jacobs, G. H., J. Neitz, and J. F. Deegan 2nd. 1991. "Retinal Receptors in Rodents Maximally Sensitive to Ultraviolet Light." Nature 353 (6345): 655–56.
Rocha, Fernando Allan de Farias, Bruno Duarte Gomes, Luiz Carlos de Lima Silveira, Sonia Limara Martins, Renata Genaro Aguiar, John Manuel de Souza, and Dora Fix Ventura. 2016. "Spectral Sensitivity Measured with Electroretinogram Using a Constant Response Method." PloS One 11 (1): e0147318.
