# Supplementary material for: Extensive topographic remapping and functional sharpening in the adult rat visual pathway upon first visual experience
Source: PLoS Biol. 2023 Aug 17;21(8):e3002229. doi: 10.1371/journal.pbio.3002229 (PMC10434970; doi:10.1371/journal.pbio.3002229)
Supplement: Attachment — Submitted filename: Response to Reviewers.pdf [file pbio.3002229.s003.pdf]

Response to Reviewers

We would like to thank all three Reviewers for their careful and thoughtful evaluation of our work, and for giving us critical feedback that enabled us to improve our work and its presentation. Thank you!

We have now addressed all the Reviewers' comments. Below, the comments are shown in **boldface**, along with our detailed responses below. Please note that in some cases, some of the comments may have been broken down for clarity and to ensure that we addressed every aspect.

Reviewer #1:

In this manuscript Carvalho and colleagues non-invasively investigated sensory remapping in the adult brain using functional magnetic resonance imaging (fMRI). Using a novel set-up allowing delivery of patterned visual stimuli anesthetized rats inside an MRI scanner, the authors succeeded for the first time in mapping receptive fields (RFs) and spatial frequency tuning curves using fMRI, hence expanding the experimental repertoire available to investigate these phenomena beyond canonical (and invasive) investigational paradigms. The authors then moved on to intriguingly track RF dynamics in visually deprived rats, revealing that light exposure can progressively promote functional remapping of unrefined visual processing systems toward functional specialization.

I found this work technically superb. The possibility of tracking remapping of visual pathways non invasively over time offers exciting opportunities for both basic and translational neuroscience.

I do not have any major criticism as most experimental confounds appear to be well controlled by these authors, who showed in this manuscript the ability to proficiently master the difficulties of fMRI in rodents. For these reasons I strongly endorse the publication of this work in Plos Biology

Thank you for your positive critique and important comments on our manuscript!

Below are very few minor questions/comments that mostly address my excitement in these methods, and my interest in promoting interpretability and repeatability of this measurements by others:

R1.1a. It would be nice to show in greater detail i.e. in a supplementary figure, a close up of the set-up when applied to the animal's head.

Thank you for the suggestion, we agree. We have now added a figure showing the visual set-up applied to an animal – new Figure S1, page 2 SI. Please also see below the new Figure!

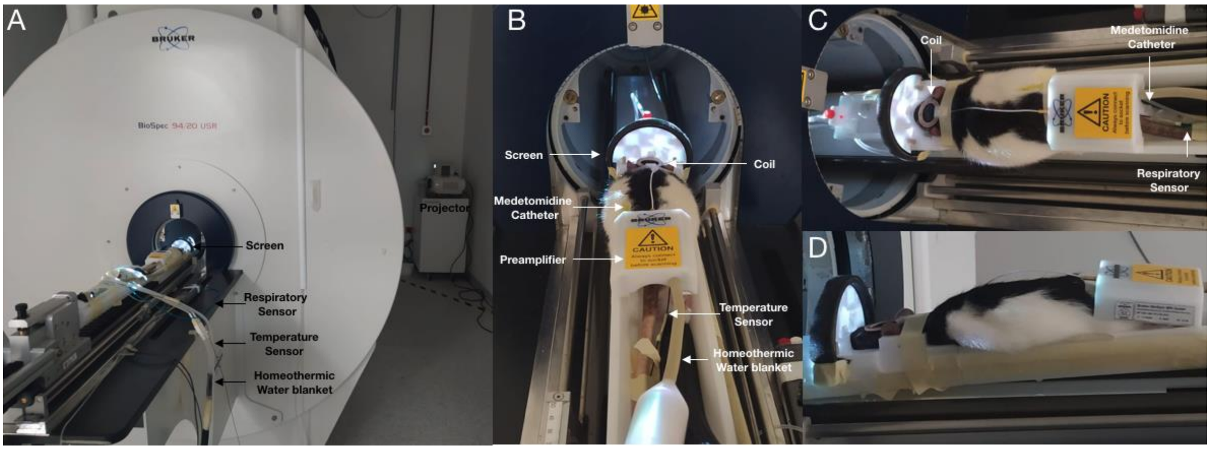

Figure R1. Photographs of the visual setup mounted to a MRI animal cradle. Animal life-support equipment: temperature and respiration sensors, intravenous catheter, and homeothermic water blanket were connected to the

cradle. The surface coil was placed on the head of the rats and the preamplifier was placed behind the animal. The visual stimulus was projected to a mirror that reflected the image to the screen placed in front of the animal eyes.

R1.1b. How could the authors achieve lack of coverage of the animals eyes, owing to the need to use a (preamplified) surface coil? A few pictures showing how the animal was arranged onto the animal cradle would be useful.

Yes, this was indeed tricky. To avoid occlusion of the Field of View, we placed the amplifier behind the animal. This is now more clearly stated on page 37 and shown in new Figure S1 (Fig. R1 above).

R1.2. Fig. S1, retinotopic stimuli: the collicular ROI chosen (D) looks slightly misplaced and potentially too small to reliably estimate the effects elicited by visual stimuli. The corresponding response is in fact spatially broader. I would probably use larger areas capturing the broader stimulated areas (panel C)?

You are absolutely correct – the depiction of the ROIs shown in Figure S1D, 3A and 3B and 4A suffered from a misalignment between the anatomical template and the underlying EPI or RARE scan. We apologize for this oversight. Please note that this was only an issue for *depicting the ROI in the Figure* and not for actually selecting the appropriate ROIs for analyses, for which we used a different template (Paxinos and Watson rat brain atlas) compared to the template we used to align our anatomical and functional images, the SIGMA atlas (Barrière et al. 2019). We had chosen to overlay Paxinos Watson atlas because it enables a detailed depiction of many rat brain structures and since it is a widely used atlas. Importantly, the functional and anatomical scans used for the analysis were properly aligned to the SIGMA atlas, therefore the misalignment does not affect the validity of our results.

Still, we completely agree that the outline of the ROIs based on the Paxinos atlas may confuse the readers. Therefore we have now outlined the ROIs based on the SIGMA atlas only. Moreover, in Figure S2 (which can also be found below, Figure R2) we have now overlaid the ROIs with the BOLD activation maps, where the ROI definition overlaps well with activated brain regions.

Regarding the superior colliculus- it is divided into superficial and deep layers. While the superficial layers have a purely visual sensory role, they receive visual inputs directly from the retina and indirectly via the visual cortex, deep layers have both multisensory (auditory, tactile or visual) and motor functions (King 2004). In this study we were purely focusing on the visual pathway and to avoid potential confounding factors with scanner noise and spurious animal movements etc, the SC ROI definition would be more appropriate if it captured the more superficial gray layers of the superior colliculus. This is likely the reason why the SC ROI is more conservative compared to the response elicited. Note that the representation of the superior colliculus using the SIGMA atlas overlays with the stronger activated region of the SC, Figure R2. Similarly the visual cortex ROI was defined as primary visual cortex (V1). In particular, in response to the spatial frequency tuning stimuli the response in the VC is spatially broader than V1, which corresponds to the activation of higher visual order (V2) areas, figure R2 . We now point this out in the methods section (pages 39 and 40).

pattern.

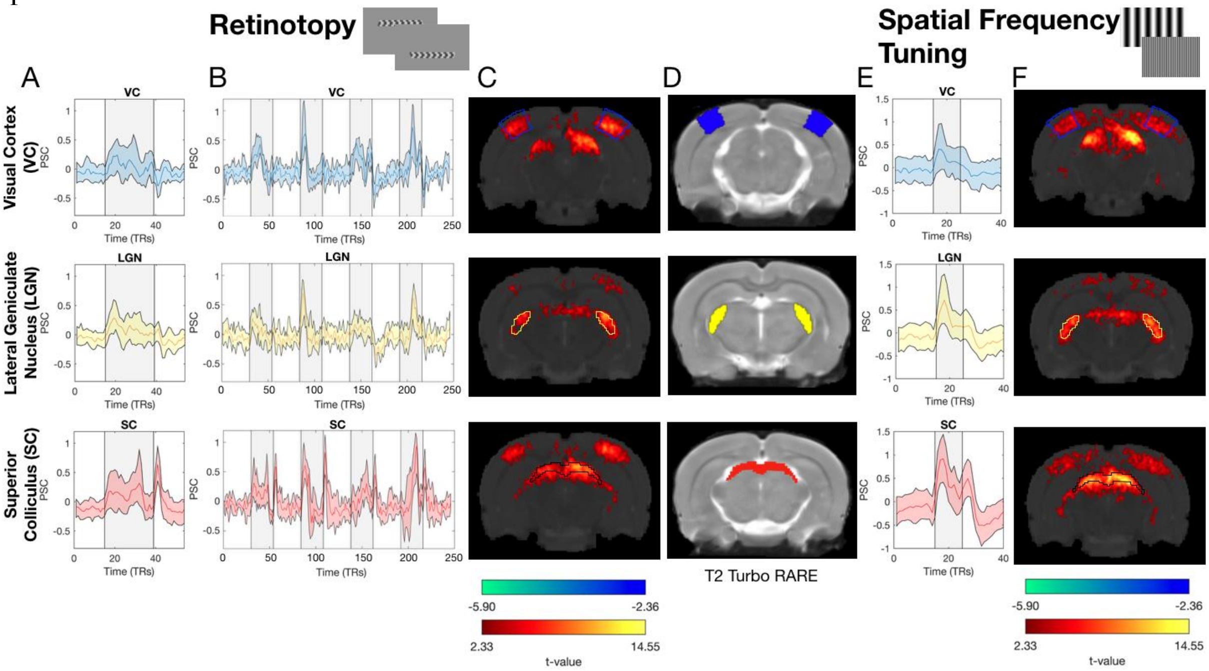

Figure R2. Retinotopic and SF tuning visual stimuli result in robust BOLD signals confined to the visual pathway in HC at t=0. A, B and E: Percentage of BOLD signal change (PSC) of the ROIs defined in D averaged across animals, runs (B) and cycles (A, E), upon retinotopic (A, B) and SF tuning (E) visual stimulation. The colored areas correspond to the 95% confidence interval and the gray area to the stimulation period. C and F: GLM functional maps obtained after retinotopic (C) and SF tuning (F) visual stimulation. The maps are FDR corrected using a p-value of 0.001 and minimum cluster size of 20 voxels. The ROIs defined based on the SIGMA atlas are overlaid on the functional maps. D: Anatomical images with the delineation of the ROIs.

R1.3. Same in Fig. 4A and S2A; here the superimposed ROI seems to straddle outside border of the brain (most likely a problem of misregistration of anatomical template to EPI underlay). This alignment should probably checked and adjusted to reflect actual ROI placement for quantification.

Thanks for pointing this out too - this has been treated exactly as in R1.2, i.e. atlases replaced for depicting the ROI more correctly, and no effect on actual ROI selection or data analyses. Figures 4A (shown below as figure R3) and S3 A now have the ROI defined based on the SIGMA atlas. These ROIs were used in the original analysis and so there are no changes in the results.

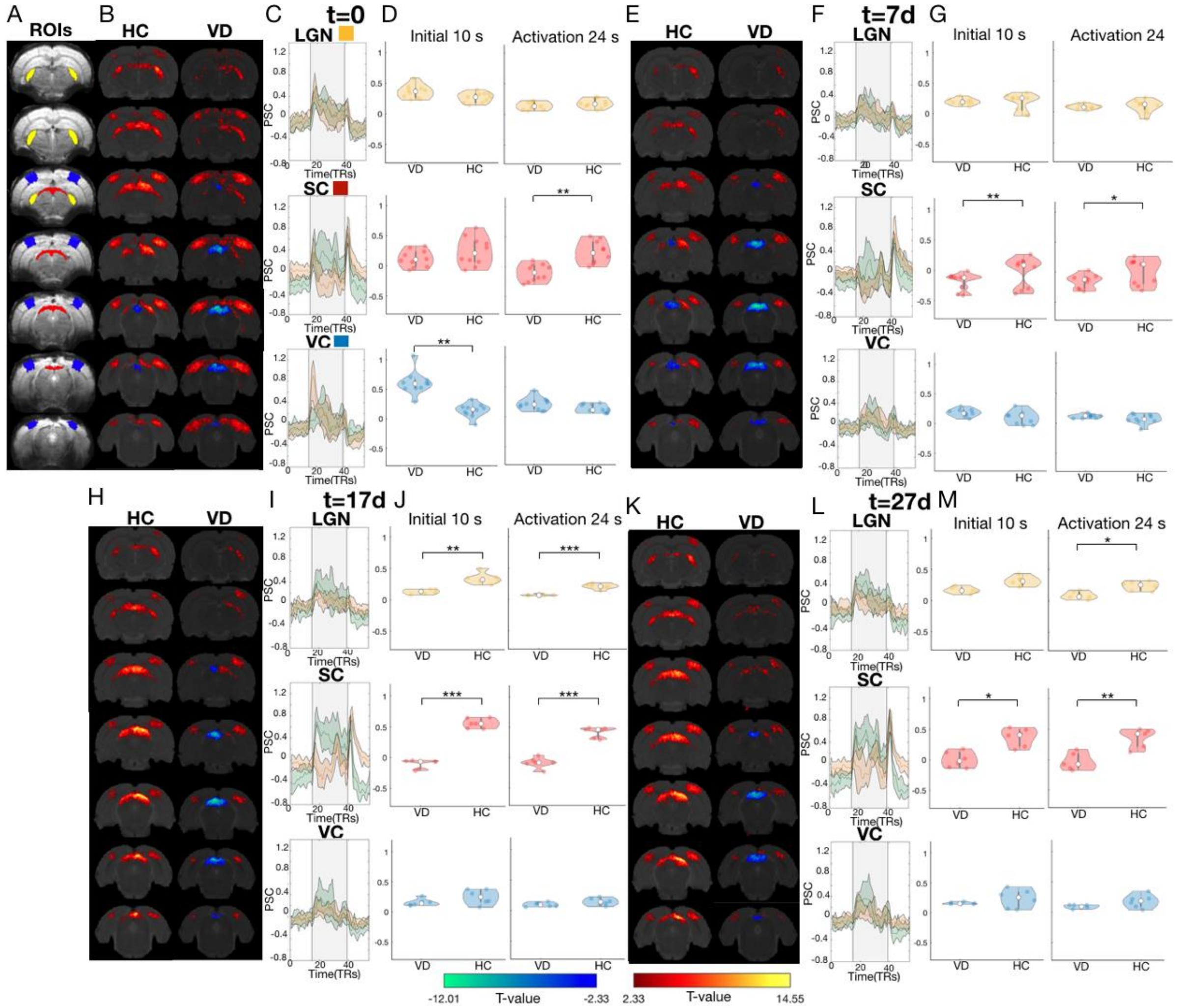

Figure R3. Differential responses between VD animals and HC driven by the retinotopic stimulus. A:Raw fMRI images with the ROIs (LGN, SC and VC) overlaid. B, E, H, K: fMRI activation patterns of t-contrast maps obtained for HC and VD animals at t=0, t=7d, t=17d and t=27d, respectively. The GLM maps are FDR corrected using a p-value of 0.001 and minimum cluster size of 20 voxels. C, F, I, L: PSC of the LGN, SC and VC for the HC and VD animals at t=0, t=7d, t=17d and t=27d, respectively. The grey area represents the stimulation period. D,G, J, M: Violin plot of the amplitude of the BOLD response of VD and HC during the initial 10 s of the activation period (left) and the total duration of the activation period obtained with the retinotopy stimulus (right) at t=0, t=7d, t=17d and t=27d, respectively. Each colored dot represents one animal. The white dot represents the mean, and the grey bar represents the 25% and 75% percentiles. The blue, yellow and red colors represent the VC, LGN and SC respectively. The *** represents a p-value<0.001, ** p-value<0.01 and * p-value<0.05. The p-values are reported in table S1.

R1.4 Analogous small misplacement in anatomical landmarking are present in Fig. 3A vs 3B. Are these functionally relevant or just a reflection of difficulties in anatomical cross-referencing of atlases?

Thanks for noticing this too, which has been treated exactly as in R1.2 and R1.3, i.e. the atlas used for the ROI visualization was replaced and now the ROIs are more accurately depicted. The misalignment between the Sigma and Paxinos Watson atlases has no influence in the ROI selection or data analyses. Figures 3A and 3B now have the ROI defined based on the SIGMA atlas. These ROIs were used in the original analysis so there are no changes to results.

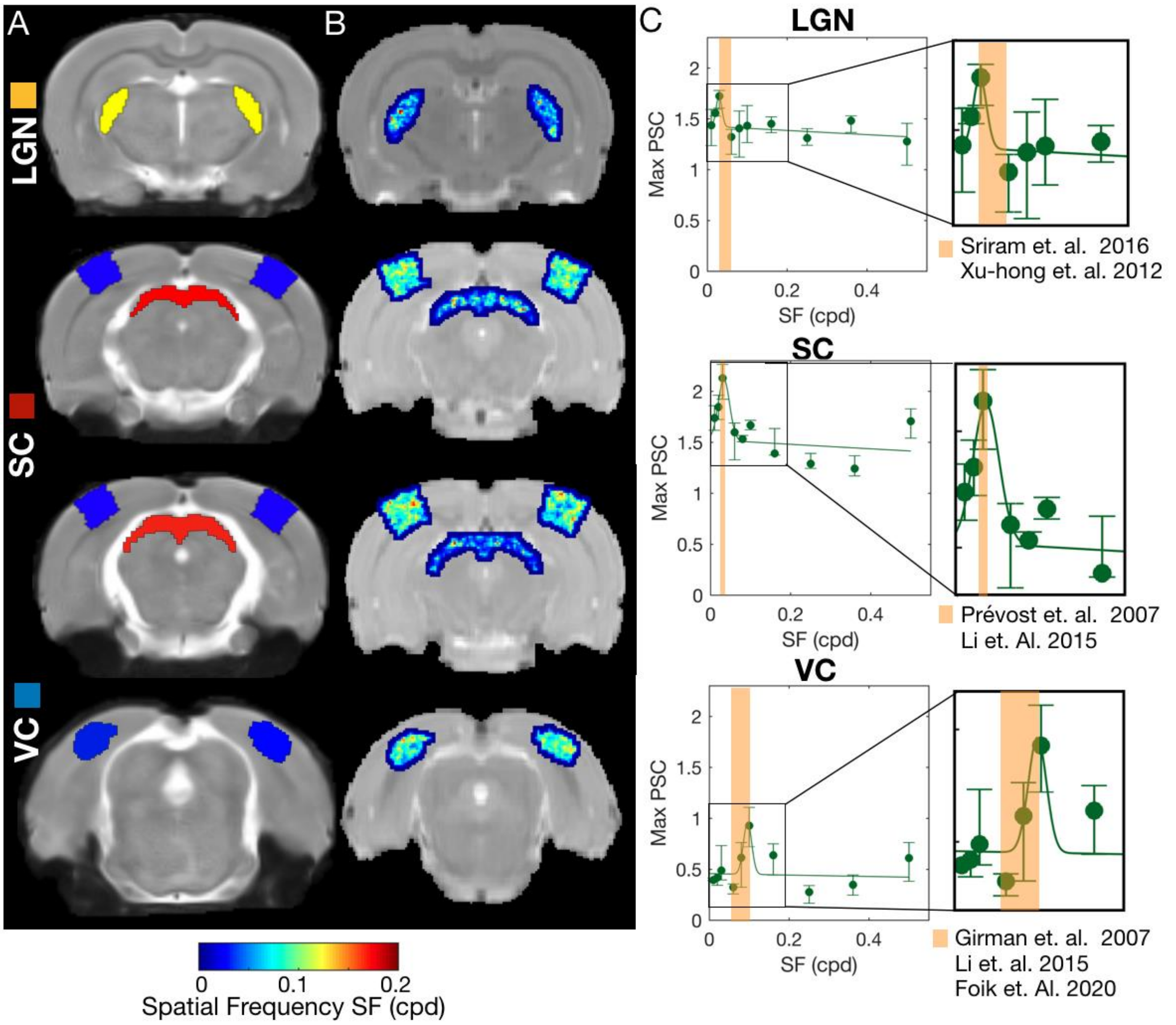

Figure R4. Spatial frequency selectivity across the visual pathway in HC animals at t=0. A: Anatomical images with the ROIs highlighted. B: Optimal spatial frequency estimated per voxel for HC, averaged across animals. C: Maximum PSC during the activation period as a function of the spatial frequency of the stimulus, calculated for HC. The errorbar represents the 10% confidence interval across animals. The continuous lines represent the Gaussian model fitted to the data. The goodness of fit is shown in Table S4. The orange band denotes the range of optimal spatial frequency values reported in the literature measured using electrophysiology. A compilation of 22 studies reporting on the optimal spatial frequency of the rat and mouse visual pathway can be found in table S3.

R1.5 I found it a bit difficult to interpret information in parametric maps in many figures, but especially Fig. 5 or Fig. 7 in which most of the space is taken up by non-relevant anatomical underlays showing brain parenchima. Perhaps a different rearrangement of the panels allowing to zoom into visually relevant regions may make the encoded information more directly interpretable to readers?

We appreciate the suggestion and agree. In response, we have readjusted Figures 5 and 7, including zoomed views of the ROIs and reorganized the panels for clarity. In Figure 5 we added a quantification of the retinotopic mapping. We trust that this is now easier to interpret and if there are any further suggestions we'd be happy to accommodate them.

Reviewer #2: In this paper authors investigated the neuroplasticity along the visual pathway using fMRI in a rodent model of visual deprivation. Using a novel setup design, they mapped in detail the topographical and neuroanatomical organization of the entire rodent visual pathway. Their data show that light exposure in adulthood results in an extensive topographical remapping and functional sharpening. Understanding the dynamics of stability/plasticity balances and how they are changed by experience is critical. Therefore, this study will have high impact on the neuroscience field. I have a few questions/comments that I hope authors can help address.

Thank you for the useful comments and for recognizing the potential of our work!

R2.1 How do researchers ensure the animal's eyes focus on the fixation point at the center of the visual field? The retinotopic mapping need to rely on this condition.

Thank you for raising this important point. We acknowledge that eye movement is a potential confounding factor in retinotopic mapping in rodents, and in fact this is quite a general point pertaining not only to our experiments but many other brain activity measurement techniques that utilize e.g. Ephys, Calcium imaging, etc.. Since we - like many others - could not directly track eye movement in this experiment, we resorted to other means to mitigate this potential confounder:

First - during scanning, the animals were sedated with medetomidine, which is widely accepted as effective in reducing spontaneous eye movements, as well as eye movements in response to visual stimuli (Garrity et al. 2015). For instance, Nair and colleagues have compared effects of common sedative drugs, and they found that ketamine/xylazine (which similarly to medetomidine is an alpha-2 adrenergic agonist (Scheinin et al. 1989; Singh et al. 2021)) reduced eye movement as effectively as 1% isoflurane with the muscle relaxant pancuronium (Nair et al. 2011). Besides being a well-established sedative for fMRI, medetomidine has shown to elicit higher electroretinogram responses than ketamine/xylazine (Connell et al. 2022). Due to the long duration of our scanning sessions and the need to have sedated animals with minimal eye movement and strong retinal responses / visual function, we chose medetomidine as the sedative.

Thus, the mitigating effects of sedation coupled to our clear observation of very reasonable retinotopic maps, suggest that eye movement confounders were significantly attenuated.

In addition, spontaneous eye movement cannot be expected to be coherent between different repetitions of stimuli. In each scanning session we performed six separate repetitions of the stimuli, and averaged them, thereby likely washing away most of any potential residual eye movement in the mean signal.

We have now elaborated on the possible confounding effects of eye movements and added the above mentioned mitigation strategy in the Discussion section (Pages 30 and 31).

R2.2 'we find no organization of spatial frequency selectivity, which indicates that each area responds globally to a set of specific spatial frequencies.' Could lack of spatial frequency selectivity be due to too coarse spatial resolution of fMRI?

Thank you for raising this important point, indeed the fact that we did not find that neighboring voxels respond to similar spatial frequencies may be related to the coarse spatial resolution of fMRI.

Whether the rodent's visual pathway exhibits spatial frequency organization (neighboring neurons respond to nearby spatial frequencies) has been under much debate, despite highly tuned visual responses. While some studies suggest that rodents present a 'salt and pepper' organization, with neighboring neurons showing a nearly random sampling of orientation and spatial frequency (Bonin et al. 2011;Ohki and Reid 2007), more recent studies have found that the similarity of spatial frequency tuning decreases as a function of cortical distance, suggesting that the rodent visual cortex is not salt-and-pepper organized (Ringach et al. 2016). In addition, it has been suggested that the lack of spatial frequency selectivity reported in other studies could be related with the resolution scale of the measurements, in particular cortical distances between 100 μm and up to 250 μm, which comprise a large number of cell pairs with disparate direction preferences. Below, we reproduce Figure R5 from (Ringach et al. 2016), which shows that the spatial frequency tuning reduces with cortical distance at multiple cortical depths. Given the spatial resolution of our acquisitions (125 × 125 x 650 μm^3) it is plausible that the absence of frequency organization in Figures 3 and 7 is due to masking by the coarse resolution of our acquisitions.

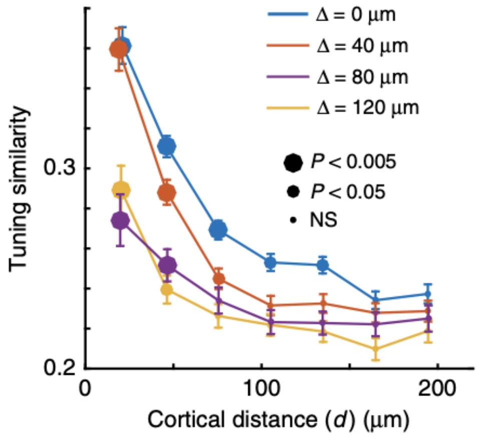

Figure R5. From (Ringach et al. 2016). Tuning similarity as a function of cortical distance and cortical depth.

We have now reformulated our initial statement that the lack of spatial frequency selectivity within visual areas indicates that each area responds globally to a set of specific spatial frequencies, to *'In contrast with the retinotopic maps, within each visual structure (ROI), we find no specific organization of spatial frequency selectivity. However, fine grained spatial frequency tuning selectivity organization might be masked by the relatively coarse resolution of fMRI acquisitions.'* (page 10). In addition, we elaborated on how fMRI resolution can potentially affect the detection of fine-grained Spatial frequency tuning properties, pages 31 and 32.

R2.3 If LGN and SC are both relays of visual information, why do they have higher sensitivity to lower spatial frequencies than VC?

Thank you for raising this important topic. To further investigate spatial frequency tuning properties across the visual pathway, we performed a small literature review of spatial frequency selectivity in mice and rats (please see Table R1 below). One of the biggest challenges that we found is that none of the studies directly compared optimal SF selectivity across the entire visual pathway. Each study focused on a particular visual area, which impairs the comparison of optimal spatial frequency selectivity across the entire visual pathway. In addition, the majority of studies used electrophysiology where only a limited number of cells

were measured. Indeed, one important advantage of our fMRI-based approach to estimate optimal SF is that we sample the entire visual pathway with the same level of specificity in the same experiment, which allows a direct and simultaneous comparison of the optimal SF selectivity across multiple visual brain areas.

From the literature, there is a high variability between the optimal SF measured for VC (0.03-0.08 cpd), LGN (0.02-0.09 cpd) and SC (0.048-0.1 cpd). One of the plausible causes of this variability is the alert state of the animals: alert mice respond to higher spatial frequencies than anesthetized mice, the specificity of the techniques used (ephys, intrinsic signal optical imaging, 2 photon imaging); and the range of spatial frequencies tested and the temporal frequency of the moving sinusoidal gratings (Andermann et al., 2011; Niell and Stryker, 2008;). Although there are less studies examining the SF selectivity in rats, the reported values seem to be more consistent between studies and very much in line with our findings: VC (0.05-0.1 cpd), LGN (0.03-0.06 cpd) and SC (~0.03 cpd), showing that VC tends to respond to higher spatial frequencies than LGN and SC.

Our optimal spatial frequency estimates link with the RF size estimated. It is assumed that there is an inverse relation between RF size and RF spatial frequency sensitivity, so that neurons with large RF sizes process mainly coarse spatial information. In our study we show that VC, which processes higher spatial frequencies, has smaller RF sizes than LGN and SC which process lower spatial frequencies. SC is thought to be the first post-retinal subcortical structure to receive visual information, therefore the low spatial frequency tuning properties of SC provide coarse (low spatial frequency) information about the visual environment, which facilitate motion detection and guiding rapid movements towards relevant targets. In addition lesion studies have shown that lesioning the SC does not impair the spatial frequency perception nor spatial acuity (Dean 1981). This suggests that high spatial frequency is predominantly processed in the cortical visual areas.

In conclusion, SC and LGN are subcortical relays of visual information, their function is not only to receive the direct projections from the rat retina and pass the information to the visual cortex where high order processing takes place, but they are involved in multiples functions related to fast responses to the visual stimulus, such as orienting the body and eye movements towards the stimulus, and guiding spatial movement using visual information (Sahibzada, Dean, and Redgrave 1986; Cooper, Miya, and Mizumori 1998; Martin 1986).

Changes to the manuscript on this topic include: (1) further elaboration on this topic in the Discussion section (pages 27 and 28); (2) addition of Table R1 to the SI (Table S3); and addition of Figure R6 to SI as well (Figure S5). We trust that this clarifies the point and again thank you for raising it!

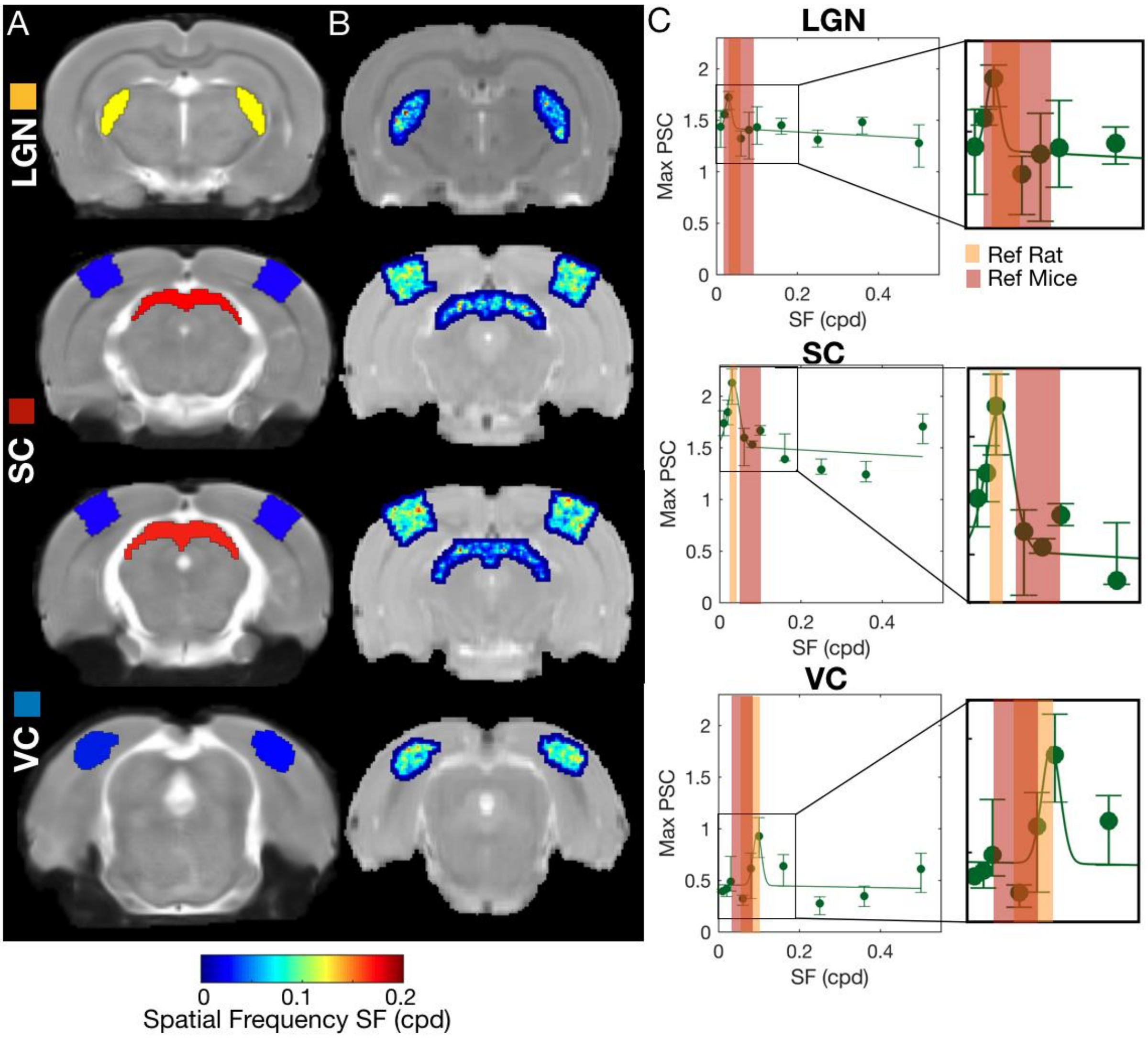

Figure R6. Spatial frequency selectivity across the visual pathway in HC animals at t=0. A: Anatomical images with Paxinos and Watson Atlas structures overlaid and the ROIs highlighted. B: Optimal spatial frequency estimated per voxel for HC, averaged across animals. C: Maximum PSC during the activation period as a function of the spatial frequency of the stimulus, calculated for HC. The errorbar represents the 10% confidence interval across animals. The continuous lines represent the Gaussian model fitted to the data. The goodness of fit is shown in Table S2. The orange band denotes the range of optimal spatial frequency values reported in the literature measured using electrophysiology. A compilation of 22 studies reporting on the optimal spatial frequency of the rat and mouse visual pathway can be found in table S4.

**Study**	**Visual area**	**Optimal SF**	**Species**	**Technique**
(Niell and Stryker, 2008)	VC	0.036 cpd	mouse	electrophysiology
(Durand et al. 2016)	VC	0.05 cpd	mouse	electrophysiology
(Gao et al. 2010)	VC	0.03 cpd	mouse	electrophysiology
(Van den Bergh et al. 2010)	VC	0.04 cpd	mouse	electrophysiology

(Zhang et al. 2015)	VC	0.046 cpd	mouse	electrophysiology Intrinsic Signal Optical Imaging
(Andermann et al., 2011)	VC	0.08cpd	mouse	2 photon imaging
(Vreysen et al. 2012)	VC	0.031 cpd	mouse	electrophysiology
(Girman, Sauvé, and Lund 1999)	VC	0.1 cpd	rat	electrophysiology
(Foik et al. 2020)	VC	0.05-0.06 cpd	rat	electrophysiology
(Li, Sun, and Shi 2015)	VC	0.068 cpd	rat	electrophysiology
(Grubb and Thompson 2003)	LGN	0.027 cpd	mouse	electrophysiology
(Durand et al. 2016)	LGN	0.08 cpd	mouse	electrophysiology
(Tschetter et al. 2018)	LGN	0.09 cpd	mouse	electrophysiology
(Piscopo et al. 2013)	LGN	0.02 - 0.05 cpd	mouse	electrophysiology
(Tang et al. 2016)	LGN	0.035 cpd	mouse	electrophysiology
(Sriram, Meier, and Reinagel 2016)	LGN	0.03-0.06 cpd	rat	electrophysiology
(Xu-hong et al. 2012)	LGN	0.04-0.05 cpd	rat	electrophysiology
(Wang et al. 2010)	SC	0.08 cpd	mouse	electrophysiology
(Ito, Feldheim, and Litke 2017)	SC	0.048 cpd	mouse	electrophysiology
(De Franceschi and Solomon 2018)	SC	0.1 cpd	mouse	electrophysiology
(Prévost, Lepore, and Guillemot 2007)	SC	0.03 cpd	rat	electrophysiology
(Li, Sun, and Shi 2015)	SC	0.03 cpd	rat	electrophysiology

Table R1. Summary of the optimal spatial frequency for rat and mice across multiple studies. The rat studies used to define the reference interval of Figure 3 are highlighted in yellow.

R2.4 Could VDM also cause alterations in vascular development, which can contribute to different BOLD responses between VD and HC animals.

Thank you for bringing up this very salient point. Indeed, already in the original version of the manuscript, we have addressed this issue by performing a hypercapnia experiment designed to investigate whether differences in vascular development can contribute to the different BOLD responses between VD and HC. We found no significant differences, please see the section in the ms. cited below:

'A total of N = 5 HC (26 runs averaged) and N=5 VD (27 runs averaged) were exposed to 1.5 min normocapnia, 1.5 min hypercapnia and 1.5min normocapnia (Figure S3). The rise times and signal amplitude nearly identical onsets between VD and HC for the different ROIs (Panels B-G). The mean PSC signal between VD and HC are highly correlated (VC: r^2=0.95 p<0.001; LGN: r^2=0.86 p<0.001; SC: r^2=0.91 p<0.001). In addition a Granger causality test showed that HC hypercapnic response is very useful to predict the VD one (VC: F=10 p-val=0.0017;

LGN: F=8.286 p-val=0.0045; SC: F=23 p-val=3.27x10^{-6}). This excludes vasculature as the major contributing factor for the different measured timing parameters. In addition the rise times and signal amplitude are nearly identical for all areas, suggesting that the vascular response dynamics becomes dissociated only at later stages.'

Currently, the auxiliary Figure associated with these results is shown in SI. If the Reviewer deems it critical to defer this Figure to the main text, we will be happy to oblige in the next round of revision.

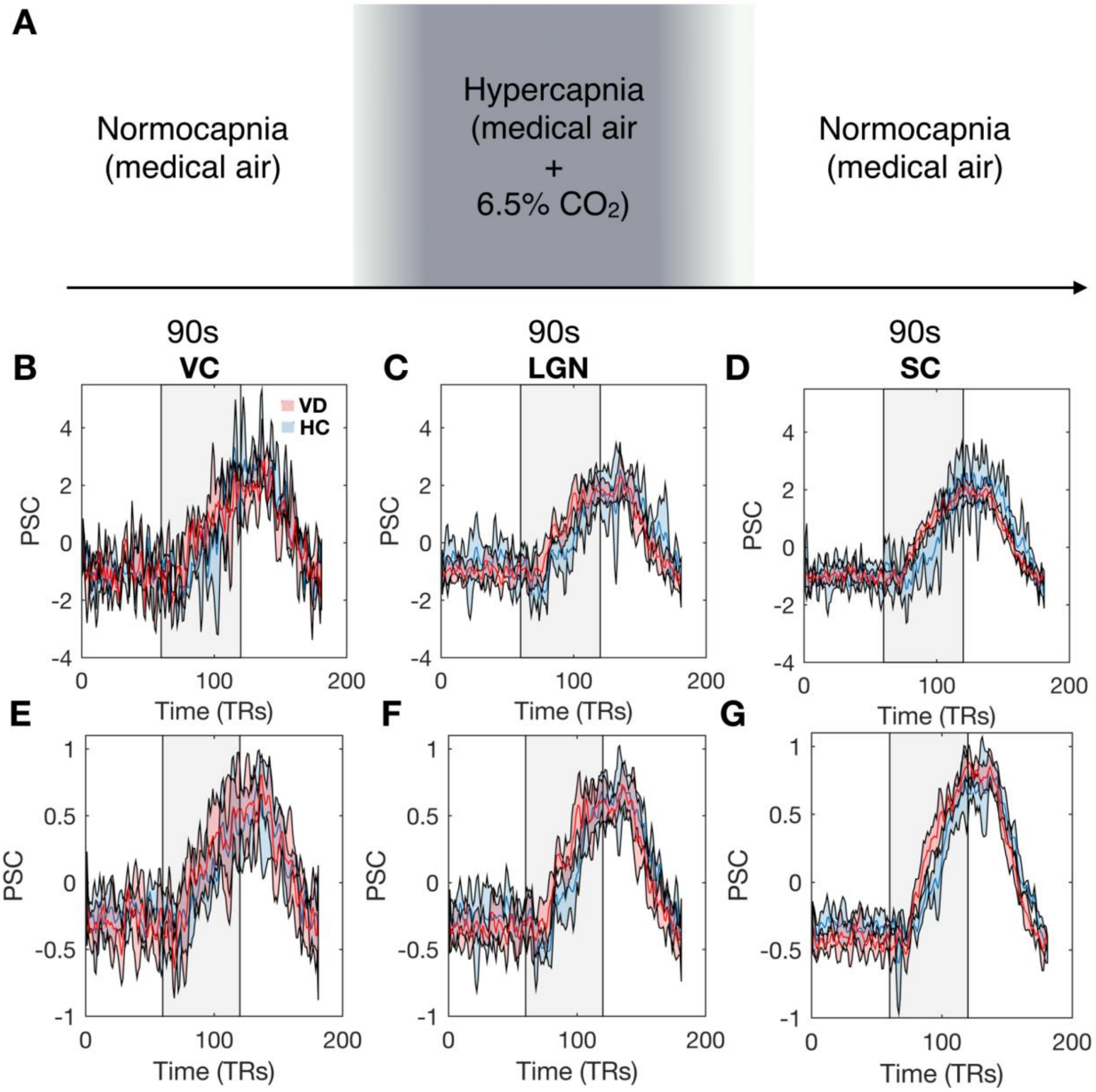

Figure R7. Hypercapnia experiment testing the dynamics of vascular responses. A: Hypercapnia paradigm consisted of a manual switch, after 1.5 minutes of medical air, to a hypercapnic state with 6.5% CO2 for 1.5 minutes. This was followed by a manual switch again to medical air for 1.5 minutes. Each run consisted in only one repetition of this block. B, C and D: PSC response profile (mean ± std) obtained for VD (red) and HC (blue) for different ROIs: VC, LGN and SC, respectively. The shaded grey area indicates the hypercapnic period. E, F and G: Normalized PSC response profile (mean ± std) obtained for VD (red) and HC (blue) for different ROIs: VC, LGN and SC, respectively.

R2.5 I suggest authors provide some quantitative assessment on the topographical remapping of pRF positions. For example, for each ROI, can spatial correlation values of RF position maps be provided between VD and HC animals at separate time points?

Thank you for this suggestion, we agree and recognize that a quantification of the retinotopic organization and the remapping was lacking in the original manuscript. In response, we have now added a quantification, based on the variation of pRF phase estimate as function of cortical thickness across the phase gradient axis defined as shown in Figure R8. In short, phase estimates were projected on the phase gradient axis and then averaged across each position of the axis. The relation between the phase and cortical distance obtained for HC and VD animals for all the scanning sessions is shown in Figure R9B. Importantly we only included in this analysis the animals that completed all 4 scanning sessions (N=6). Figure R9B shows that while HC animals show that phase varies with cortical distance consistently across all scanning sessions, for VD animals at t=0 the relation between pRF and cortical distance is nearly random, which is evidenced by the flat slope. With light exposure the relation between pRF phase and cortical distance becomes more apparent and the slope progressively increases in magnitude (Figure R9B). Figure R9C shows that at t=0 the magnitude of the slope between phase variation and cortical distance is significantly higher for HCs than for VDs for VC, LGN and SC. Whereas at t=27d HCs and VDs show nearly identical slopes for all visual areas. The quantification of the retinotopic organization of the visual pathway confirms that visual exposure even post critical period promotes the organization and specialization of the visual pathway.

In addition, we applied a complementary approach to quantify the retinotopic arrangement of the visual pathway across scanning sessions. This approach does not only take into account the pRF phase but also its eccentricity and size. Here we looked at the pRF profiles estimated with micro probing technique (Figure R10A) and we computed the similarity between pRF profiles (Figure R10C) and the cortical distance between every voxel of each ROI (Figure R10B). We can see that for HC the similarity between pRF profiles is anticorrelated with the cortical distance and it is consistent across scanning sessions (t=0 r^2=-0.31; t=7d r^2=-0.29; t=0 r^2=-0.29; t=7d r^2=-0.28; Figure R10C), showing that neighboring voxels have more similar pRF profiles than distant voxels. On the other hand, at t=0 for VD animals shows little to no correlation between the pRF similarity coefficient between cortical distance (t=0 r^2=-0.14). With visual exposure the VD pRF similarity coefficients become more similar to those of HC and progressively more anticorrelated with cortical distance (t=0 r^2=-0.14; t=7d r^2=-0.16; t=0 r^2=-0.2; t=7d r^2=-0.26; Figure R10C). This approach further confirms the results of the phase gradient analysis and shows that light exposure drives a progressive reorganization of the visual pathway.

Given these important augmentations, we replaced Figure 5 for Figure R9 in the main manuscript and we added Figure R8 and R10 to supplementary material. We discuss the findings of panels 5B and 5C on page 15 and in the Methods section we detail the retinotopy quantification analysis, pages 41 and 42.

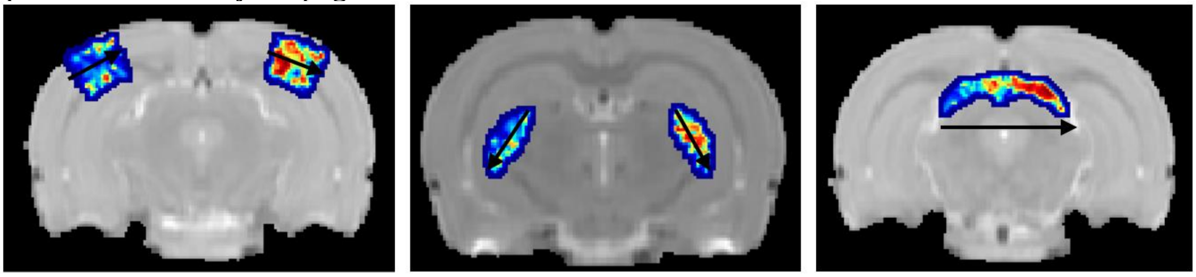

Figure R8. Gradient direction for VC, LGN and SC.

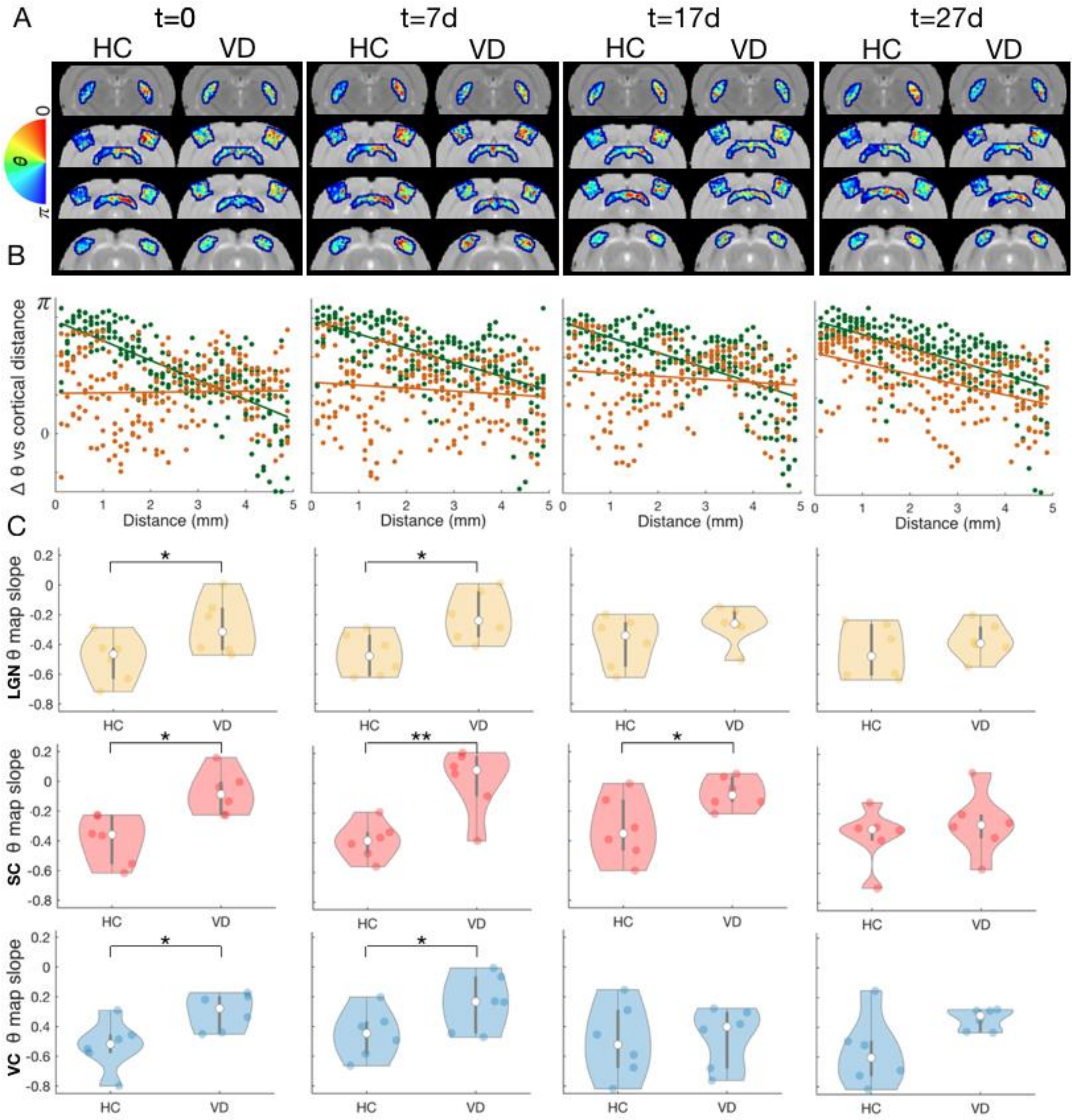

Figure R9. Refinement of RF position across time for VD and HC. A-D: Average phase maps obtained for 4 different slices obtained for VD and HC at the 4 measured time points (t=0, t=7d, t=17d and t=27d). B Variation of the pRF estimated phase as function of the cortical distance across gradient direction, measured for the SC of HC (green) and VD (orange) across multiple time points (t=0, t=7d, t=17d and t=27d). C Violin plot of the slope of the correlation between the pRF phase variation and cortical distance measured for HC and VD, for each ROI (LGN, SC and VC) for multiple time points (t=0, t=7d, t=17d and t=27d). Each dot corresponds to a different animal. Only the animals that performed the four scanning sessions were included in the analysis. The *** represents a p-value<0.001, ** p-value<0.01 and * p-value<0.05.

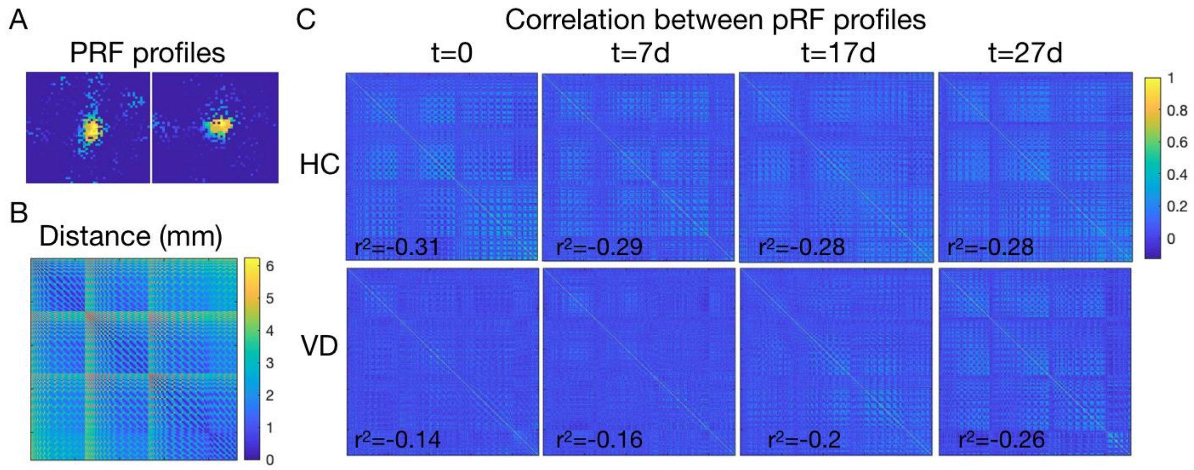

Figure R10. Quantification of the topographical organization of the visual pathway. A: pRF profiles of 2 adjacent pRFs. B: Distance matrix of all the SC voxels. C: PRF similarity analysis of all SC voxels averaged for all the animals.

<u>Reviewer #3</u>: This paper addresses a problem of general interest. It is technically sound and well written and its conclusions appear in general justified. Still there are some areas in the paper where further clarifications would be useful to the reader and would enhance confidence in the results:

Thank you very much for the positive feedback and important comments on our manuscript!

Comment R3.1. Figure 3 is a bit surprising in that the peak of the frequency response appears to be quite narrow, more so that one would expect from electrophysiological or psychophysical studies in the rodents. Perhaps this narrowness is an artifact of the fit or a technical issue with fMRI imaging normalization, but it would be nice to comment on it and offer an explanation.

Thank you for the important comment – we completely agree and clarify below.

The range reported in yellow corresponds to the optimal spatial frequency reported by <u>multiple</u> electrophysiology studies in rats, using different methods and under different physiological conditions. This explains the range of values from the various studies that we report, rather than a particular single value. This breadth may indeed give an impression that the fMRI-based spatial frequency tuning curve is narrower than what has been estimated using Ephys, 2 photon imaging and intrinsic optical imaging, but this is likely due to the varying conditions of the other studies rather than reflecting any narrower responses in our study. The high variability in other studies is likely due to multiple factors, for example alert state of the animal (anesthesia reduces the optimal spatial frequency detected, the range of spatial frequencies tested, the temporal frequency of the moving sinusoidal gratings and the different techniques used: Ephys, 2 photon imaging and intrinsic optical imaging). Naturally, the values reported for each individual reference study are narrower than the interval shown in Figure 3. To more accurately compare our spatial frequency tuning properties to previous invasive studies, we summarized the results of SF selectivity of 22 studies (Table S3) and we added a Figure similar to Figure 3 where we added the mouse spatial frequency reference values (Figure S5). In addition, in Figure 3, we added the studies used to define the reference interval so that it is clear where the interval actually comes from.

Nonetheless, we do agree with the Reviewer that there are multiple factors that can contribute to the narrower fMRI spatial frequency tuning curves: **(1)** they were obtained from the signal of the entire ROI. The spatial frequency tuning curves shown on Figures 3 and 7 were obtained by calculating the optimal spatial frequency on the averaged ROI signal during the activation period of each individual activation block. As depicted in Figure 3, the baseline of the tuning curves remains high throughout frequencies suggesting that there are responsive voxels to all the spatial frequencies and the width of the tuning curve correspond to the spatial frequency interval to which there is stronger overlap between the response of more voxels; **(2)** the coarse spatial frequency sampling (only 10 SF were tested, having more points between 0.03 cpd and 0.12 cpd could have improved the fits); **(3)** N=2 runs of the spatial frequency stimulus were averaged; and **(4)** the inherent courser resolution in fMRI can also affect the SF tuning fit. We now have elaborated on these extra limitations of the fMRI approach to estimate spatial frequency tuning curves in the Discussion section (pages 27 and 28). We have further updated Figure 3 referring to the studies used to benchmark the fMRI based estimated spatial frequency tuning curves.

Comment R3.2 In figure 4, the number of animals listed in the methods seems is 10 but the number of points in the violin plots is greater (perhaps 20). I assume this is because slices are plotted rather than animals. Although the results look significant, it would be

important to report whether statistical comparisons involve comparison across animals rather than slices and whether they are affected by multiple comparison corrections (was the multiple comparison across the 3 areas or also time points?).

We completely agree. Indeed Figure 10 deserved a better explanation. Each dot represents a stimulation block, per animal there are 4 stimulation blocks. We have modified Figure 4 so that every dot represents an animal (Figure R1). We moved the initial version of Figure 4 to SI, the shape of the violin plots is very similar between the two figures, suggesting that for each animal the activation of the stimulation is consistent between blocks. Nonetheless, the statistical analysis was done across animals. We have repeated the statistical analysis, based on an Anova, post hoc analysis Bonferroni corrected for multiple comparisons (for the measurements: brain areas and sessions). The p-values are reported in Table S1. Except LGN during the 24s activation period which lost its significance, all the significant differences between HC and VD were maintained with this more stringent statistical analysis.

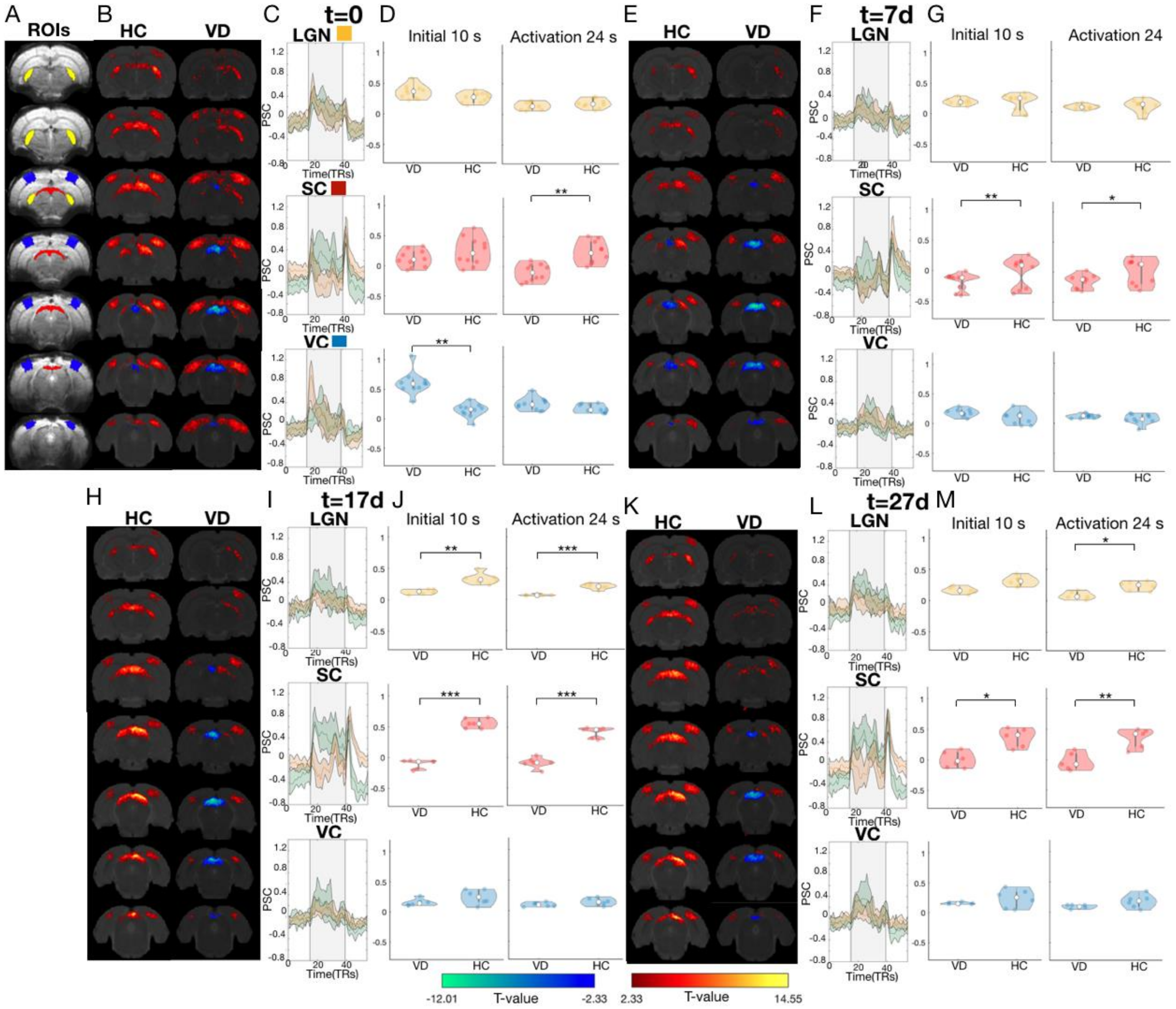

Figure R11. Differential responses between VD animals and HC driven by the retinotopic stimulus. A: Raw fMRI images with the ROIs (LGN, SC and VC) overlaid. B, E, H, K: fMRI activation patterns of t-contrast maps obtained for HC and VD animals at t=0, t=7d, t=17d and t=27d, respectively. The GLM maps are FDR corrected using a p-value of 0.001 and minimum cluster size of 20 voxels. C, F, I, L: PSC of the LGN, SC and VC for the HC and VD animals at t=0, t=7d, t=17d and t=27d, respectively. The grey area represents the stimulation period. D,G, J, M: Violin plot of the amplitude of the BOLD response of VD and HC during the initial 10 s of the activation period (left) and the total duration of the activation period obtained with the retinotopy stimulus (right) at t=0, t=7d, t=17d and t=27d, respectively. Each colored dot represents one animal. The white dot represents the mean, and the grey bar represents the 25% and 75% percentiles. The blue, yellow and red colors represent the VC, LGN and SC respectively. The *** represents a p-value<0.001, ** p-value<0.01 and * p-value<0.05. The p-values are reported in table S1.

R3.3 In the description of figure 4 in the text there appear to be several apparent description mistakes. For example, in line 198 - "in the first 10s of visual stimulation .... the bold response in VC and LGN in the VD group was significantly higher than in HC (fig. 4C,D)" -- however in figure 4C,D the groups is labeled as DR and there does not appear to be significant difference in LGN. In line 210-11 "for all time points, the VD SC exhibited a negative bold response to the retinotopic stimulus..." but not all the mean SC responses in the figure appear to be negative. I recommend aligning the labels (VD, DR) between the text and figure and reviewing the text description more carefully to avoid inaccuracies. Also it would be good to review abbreviations and ensure they are defined during the first time of use.

Thank you for spotting these oversights, which (mainly) came from prior drafts of the manuscript. All issues have now been corrected, the legend aligned, now we only use VD not DR, and we reduced the abbreviations throughout the manuscript as much as possible.

We mentioned that "*for all time points, the VD SC exhibited a negative bold response to the retinotopic stimulus*..." referring to the interpretation of the BOLD activation maps which show negative BOLD response in the SC in all time points, also the time courses shown in the panels C,F, I and L show that for all the time points the initial overshoot is followed by negative signal, contrary to the responses in HC that maintain its positiveness. SC is a key player in saliency detection and its BOLD signal has three distinct phases: an onset, a steady-state phase and an offset (Gil, Valente, and Shemesh 2022). In Figure 4D, G, J, M we averaged the BOLD signal across the entire stimulation, merging the initial phases of the SC signal (onset and steady-state). This is why not all mean SC responses appear to be negative as pointed out, we have now clarified this, in the Results section (Pages 12 and 13).

Furthermore in a recent study Gil et al, investigated the continuity illusion phenomenon in the rat through behavior, fMRI and electrophysiological recordings. They found that when continuity illusion is achieved, the SC fMRI signal changes from positive to negative regimes. Importantly electrophysiological recordings in SC point to neuronal suppression as a source of the negative BOLD signal (Gil, Valente, and Shemesh 2022). This suggests that chronic visual deprivation disrupts the activation-suppression balance in SC, leading to negative BOLD responses during the steady-state phase in the SC in VD animals throughout the four scanning sessions. This is now discussed in detail in the Discussion, page 25.

R3.4 Line 177 -- peak spatial frequency response in visual cortex I believe should be at 0.1 cpd not 1 cpd

You are absolutely correct, thank you for the sharp eye! We have now corrected this oversight.

R3.5 In figure 6, it would be helpful to make a statistical comparison of the phase maps across animals to quantitatively assess the significance and magnitude of the observed changes.

The Reviewer is probably referring to Figure 5, and we completely agree that a quantification of retinotopic mapping and topographic remapping was missing the original version of the manuscript.

In response (also to R2.5), and despite that such quantifications are actually very rare in the literature, we have implemented two complementary approaches to quantify the retinotopic

organization over scanning sessions between HC and VD animas. A full description can be found above in our response to R2.5. Briefly we added a quantification of the phase variation with cortical distance to Figure 5. Figure R5.B shows that phase varies with cortical distance consistently across all scanning sessions, for VD animals at t=0 the relation between pRF and cortical distance is nearly random, which is evidenced by the flat slope. With light exposure the relation between pRF phase and cortical distance becomes more apparent and the slope progressively increases in magnitude (Figure R5B). Figure 5C shows that t=0 the magnitude of the slope between phase variation and cortical distance is significantly higher for HCs than for VDs for VC, LGN and SC. Whereas at t=27d HCs and VDs show nearly identical slopes for all visual areas (pages 15 and 16).

R3.6 Line 254 --" HC pRF estimates for VC LGN and SC did not significantly differ between scanning sessions" -- In figure 6A for LGN it appears that the difference between t=0 and t=7 d for HC (green) might actually be significant -- would it be possible to re-check-- Also the error bars do not appear to reflect standard deviation as stated in the legend (see for example the brown bar, LGN, t=27d). It would be good to state in the legend whether the statistical tests were done across animals and whether multiple comparisons were implemented or not. Was ANOVA used or pairwise t-tests?

Thank you for pointing this out. We repeated the pRF size analysis including rerunning the statistical analysis using the most stringent Bonferroni correction for multiple comparisons (brain area and session). The p-values are detailed in table S2 (Supplementary information). Indeed some error bars were reflecting the standard error of the mean instead of the standard deviation, this is now corrected, and we also confirmed that the error bars in Figures 7C and 7D reflect the standard deviation. With this new, more stringent, statistical analysis, some of the p-values are (as expected) somewhat higher but all the significant differences reported in the original version of the manuscript still survive the Bonferroni correction at 0.05 level of significance ($p<0.05$). Indeed, the pRF changes obtained for the LGN in HC from t=0 to t=7d became significant, and we have now acknowledged this in the Results section. We have updated Figure 6 with the new plots, and added the information regarding the statistical analysis to the legend and Methods section (page 43).

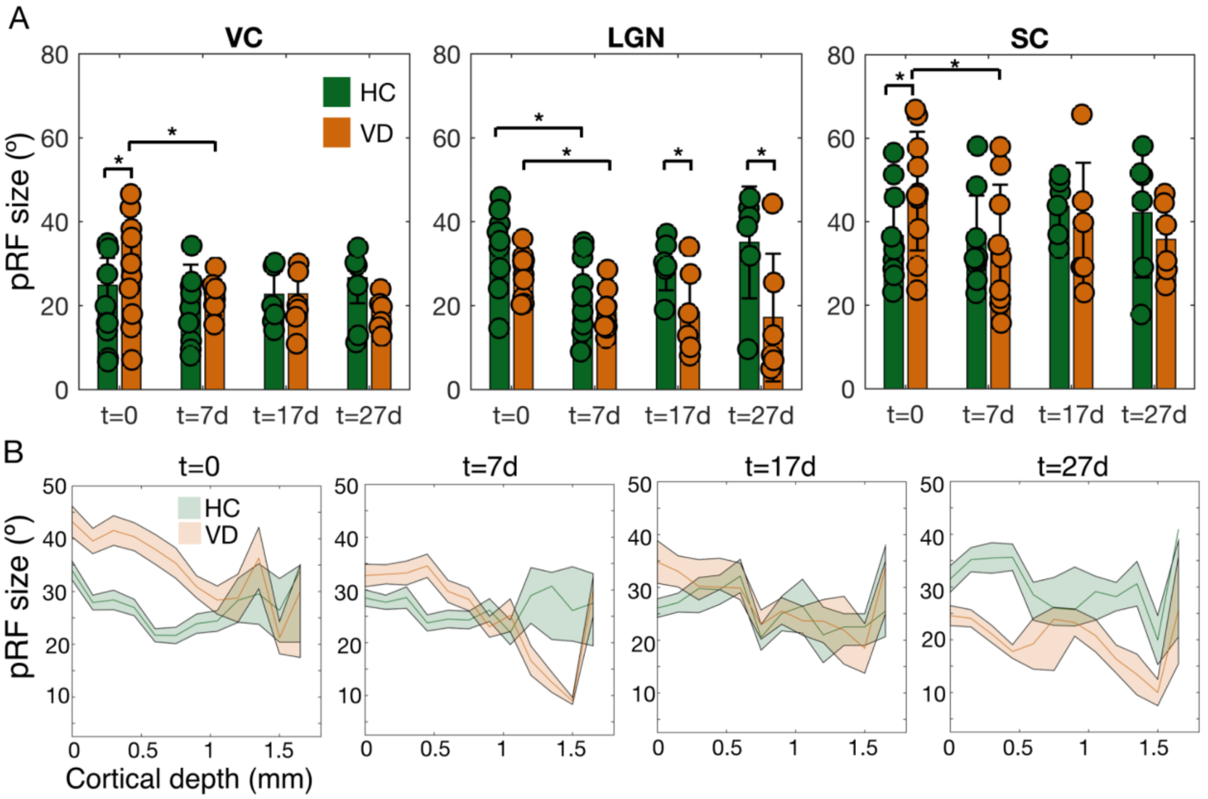

Figure R12. Refinement of RF size across time for VD and HC. A: Average RF size measured for HC and VD at multiple time points for VC, LGN and SC. The errorbar corresponds to std. The *** represents a p-value<0.001, ** p-value<0.01 and * p-value<0.05. The statistical analysis was performed using ANOVA Bonferroni corrected for multiple comparisons (brain area and scanning session. The p-values are detailed in table S2. B: Variation of the RF size averaged across animals as a function of the cortical depth for two slices of the VC for VD and HC at multiple time points.

R3.7a The example given in 6B does not appear to be the best example of the evolution of pRf sizes seen in line 6C. The color map represents variance explained but is this done by micro-probing or by conventional prf mapping?

The visualization of the pRF profiles shown in Figure 6B is obtained using the micro probing technique and indeed the color map shows the variance explained which gives a visualization of an individual pRF profile. We agree that panel 6B is complicated to interpret in terms of the evolution of the pRF size over time and for this reason we removed it from Figure 6.

R3.7b What is the variance explained that would be corresponding to noise -- ie from voxels in an area that is not visually responsive?

The "variance explained" of voxels in non visual areas is a very relevant issue. In response, we have calculated the pRF in the Auditory cortex (AC), Motor Cortex (MC) and Basal Ganglia (BG) for the HC and VD animals for t=0. As expected the Variance Explained is the visual areas is higher than the VE in non visual areas (~0.03), note that in our analysis we have only considered the pRF whose variance explained is above 0.05, this excludes very noisy voxels and it is well above the variance explained of the pRF is non visual areas. The number of voxels excluded can be found in table S3. Moreover, the pRF profiles obtained for AC, MC and BG provide a good visualization of how noisy without a defined pRF are the measurements in areas that are not visually responsive, Figure R13. We have added figure R13 to the SI (Figure S8) and we elaborated on this topic in the Results section, page 8.

All these analyses further strengthened our conclusions and we thank the Reviewer for the suggestion.

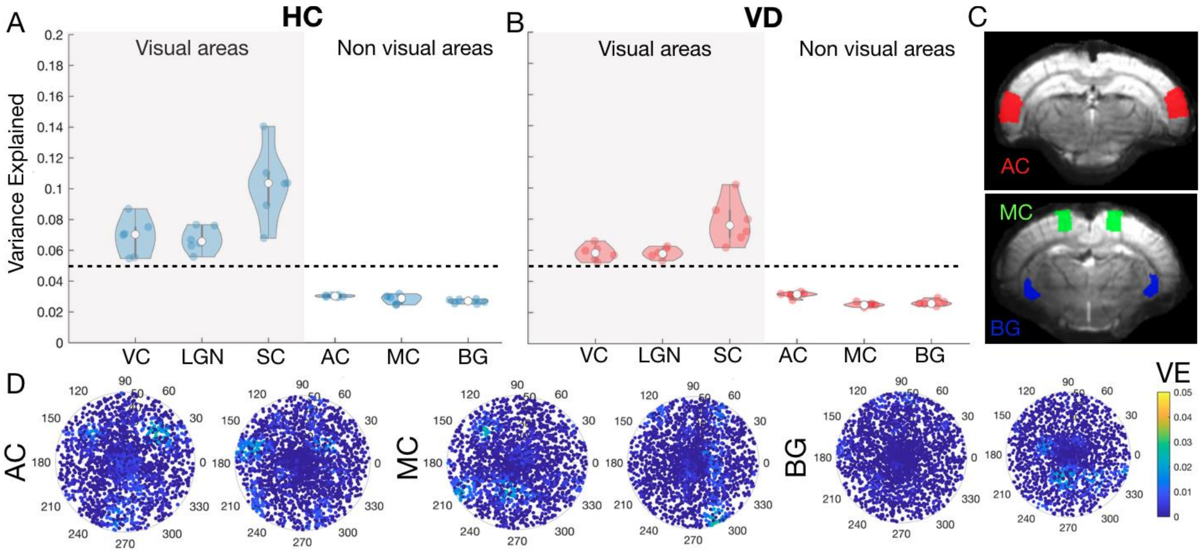

Figure R13. PRF estimates in the AC, MC and BG. A and B: Violin plots of the variance explained calculated for visual areas (VC, LGN and SC) and for areas not visually responsive (AC, MC and BG) for HC and VD, respectively. C. Anatomical image with the ROIs AC, MC and BG overlapped. D: PRF profiles obtained for HC and VD animals for AC, MC and BG.

R3.8a In Figure 7: is time point 17d reliable? It appears very little activity is seen in VC for this animal. Is this physiological or is it possible that it represents experimental/technical variability.

Thank you for raising these very valid points. Indeed at t=17d and t=27d there is very little activity in the VC for controls and also in visually deprived animals as response to the SF stimulus, as clearly shown in Figure S2. We have reasons to believe that the lack of / diminished activity in VC at t=17d and t=27d is reliable and reflects adaptation to this particular stimulus, rather than being a bias from the experimental set-up, acquisition, visual stimulus, physiology, or analysis. We briefly outline the reasons below:

First, during the same sessions (t = 17d and t = 27d) the animals exhibited clear activation in the visual cortex in response to the retinotopic stimulus (Figure 4). Apart from the VD animals that exhibit a reduction in BOLD activation in the VC from t=0 to t=7d, the BOLD signal in the VC did not vary between sessions, see Table S2. This shows that during the same scanning session the VC is responsive to retinotopic stimulus and remains consistent across all scanning sessions. Given that we performed all the experiments in the same sessions using the same set-up for the display of the spatial frequency stimulus and retinotopic stimulus it is unlikely that the lack of BOLD response to SF stimulation represents experimental/technical variabilities across time.

Second, the animals were induced using isoflurane, which is a vasoactive drug that affects the basal BOLD signal (Tsurugizawa, Takahashi, and Kato 2016). As the spatial frequency stimulus was presented after the retinotopic stimulus, the remaining isoflurane effects should have been washed away during scan preparation and anatomical image acquisitions, and therefore are highly unlikely to be the responsible for the lack of BOLD signal in SF stimulation (while in the retinotopic stimulation, as mentioned above, the BOLD responses were preserved).

Third, the spatial frequency stimulus was isoluminant across conditions, the only difference is that in each run of the spatial frequency stimulus the order of SFs was

pseudo randomized, but the stimuli characteristics did not change between sessions. Therefore, this cannot explain variability between sessions.

Fourth, the fact that LGN and SC show clear activation in the same experiment in which the VC does not exhibit strong activation also proves that the visual stimulation was properly delivered and that the animal physiology was stable. If LGN and SC were only responding to luminance changes instead of spatial frequency, we should expect that the strength of the BOLD response would be constant across spatial frequencies; however we see that some spatial frequencies elicit a stronger BOLD response. Thus, the setup was correctly working and the animal physiologically stable.

Fifth, although the signal is highly reduced in the VC in Session 3 and therefore noisy, it still peaks at the spatial frequency values that correspond to the optimal SF selectivity for VC, again indicating that the few neurons that are active maintain spatial frequency selectivity.

Taken together, these points strongly suggest that plasticity or adaptation to the stimulus underlies the lower BOLD modulation in VC in these time points. This effect needs to be investigated in future studies and was clearly beyond the scope of this one, but it highlights the power of using fMRI for investigating the whole pathway using our novel setup.

Although we have no valid reason to exclude these data points, we agree that the fact that we have low BOLD amplitudes for the VC at those time points reduces the contrast to noise ratio which can affect the reliability of the fit to spatial frequency tuning curves of VC at t=17d and t=27d, we now point out this limitation in the discussion section. Nevertheless, this does not affect the main findings of the manuscript from t=0 to t=7d we see that the spatial frequency tuning curves in VC become more similar between controls and visually deprived animals.

We now discuss this in the Results and Discussion sections, pages 28 and 29.

R3.8b It also appears the LGN, SC responses are not just responses to spatial frequency but to luminance as the baseline remains high throughout. Was the stimulus chosen isoluminant across conditions? If so what is causing the PSC to stay high across SF conditions for LGN and SC?

There are multiple reasons contributing to the baseline remaining high. First, there are responsive voxels to all the spatial frequencies tested and neurons in VC, LGN and SC show a high variability in the spatial frequency tuning properties. In particular the spatial frequency cut off (a measure of the spatial resolution) in the rat LGN, SC and VC varies between the following intervals ([0.01 and 1 cpd]; [0.02 1.2 cpd]; [< 1.2 cpd]) (Sriram, Meier, and Reinagel 2016; Prévost, Lepore, and Guillemot 2007; Girman, Sauvé, and Lund 1999), this covers the entire range of spatial frequencies that we used to estimate the tuning curves. Therefore, the fMRI based spatial frequency tuning curves shown in this study correspond to the spatial frequency interval to which there is stronger overlap between the voxels response (spatial frequency range that most voxels respond strongly). Second, LGN and SC are both relay regions of the visual system, mediating the two main visual pathways: the geniculate pathway (via LGN) and the extrageniculate pathway (via SC). In particular SC is highly involved in saliency/luminance detection and response habituation (Gil, Valente, and Shemesh 2022). SC and LGN neurons respond mainly to high-contrast moving stimuli and they are involved in high speed visual information processing crucial to orienting the body and eye movements towards the stimulus, and guiding spatial movement using visual information (Prévost, Lepore,

and Guillemot 2007); (Sahibzada, Dean, and Redgrave 1986; Cooper, Miya, and Mizumori 1998; Martin 1986; Foik et al. 2020).

All of these points are now discussed in the manuscript, page 19.

R3.9a It would be good to discuss whether the refinement of pRF responses is due to single cell receptive field refinement versus decrease of receptive field variability across neurons that belong in the same voxels.

Thank you for raising these excellent points. One of the limitations of using fMRI is that it of course lacks single cell specificity so experimentally it is not possible to disentangle whether the pRF refinement arises from single cell RF refinement or from a decrease in RF variability across the population of neurons belonging to the same voxel. In our view the two possibilities are also tightly interrelated. Previous single-cell studies have shown that visual RFs and retinotopic maps develop through individual cell refinement processes driven by fine-scale circuit refinement in which imprecise connections are weakened and eliminated and correctly targeted connections are strengthened and maintained, leading to the maturation of surround suppression in visual neurons (Katz and Shatz 1996; Pecka et al. 2014). At a population level the refinement of individual RFs will lead to a decrease in variability of the RFs properties of neurons captured by the same voxel. We have now discussed this in the manuscript, page 32.

R3.9b Also it would be useful to discuss more specifically why the experimental differences between the present study and reference 33 would explain how results differ.

There are multiple factors that contributed to the different results between this study and the of the Gil et al 2021, in summary: **(1)** dramatic differences between the delivered stimulus; **(2)** experimental design; and **(3)** species used.

Different stimuli lead to stimulation of different subpopulations of visual neurons specific to different visual properties. Human fMRI studies have shown that varying the retinotopic stimulus properties lead to different pRF estimations due to the targeting of different neuronal populations within a voxel(Yildirim, Carvalho, and Cornelissen 2018). In Gil et al 2021, the stimulus consisted of a flickering blue LED (only two flickers were delivered). In our present work, we presented far more complex stimuli, i.e the retinotopic and spatial frequency stimuli consisted of high-contrast moving stimuli moving in multiple directions and orientations, which can activate different visual areas. In particular it has been shown that neurons with the visual pathway have a preference for high-contrast moving stimuli, in particular SC and LGN, direction and orientation changes (LGN , VC). Furthermore, in Gil et al 2021, the stimulus presentation was very brief (1 second of visual stimulation followed by 40s baseline), whereas in the present study, each stimulation block lasted for 24s (retinotopy) and 15s (spatial frequency) followed by 45 s of baseline acquisition. Given the role of some visual areas (SC) in response habituation/ novelty detection and the cumulative nature of BOLD responses, we expect highly different BOLD responses between the two studies. In a more recent study, Gil et al. 2022 varied the temporal flickering frequency of the LED visual stimulation, which resulted in dramatic changes in the BOLD response profile across the entire visual pathway (including the changes in the positiveness of the BOLD responses). The fact that the change of a single stimulus parameter can result in such dramatic changes in the BOLD responses suggests that the different findings between this study and the one of Gil et. al. 2021 can be attributed to the different types of stimulus used. We believe that more complex and well

defined stimulus with longer duration will elicit more reliable BOLD responses, and they are better suited to probe plasticity mechanisms.

In addition to the stimuli, the experimental designs of the two stimuli are very different. Gil et al 2021 study was designed to measure activity onsets using ultrafast (single slice) BOLD acquisitions. This study is experimentally very different, as it was designed to assess timing properties reflecting plasticity changes across the visual pathway, in particular the number of slices (12 vs 1), the TR (1.5 s vs 50 ms) and the slice orientation.

Lastly, the two studies used different species, Gil et.al. used mice and we used rats, which we already showed to present significant differences in the processing of visual information (Figure R3) and respond to anesthesia differently.

We have now elaborated on the possible sources for the differences between our study and Gil. et al.2021 in page 23 and 24.

REFERENCES

Barrière, D. A., R. Magalhães, A. Novais, P. Marques, E. Selingue, F. Geffroy, F. Marques, et al. 2019. "The SIGMA Rat Brain Templates and Atlases for Multimodal MRI Data Analysis and Visualization." *Nature Communications* 10 (1): 5699.

Bonin, Vincent, Mark H. Histed, Sergey Yurgenson, and R. Clay Reid. 2011. "Local Diversity and Fine-Scale Organization of Receptive Fields in Mouse Visual Cortex." *The Journal of Neuroscience: The Official Journal of the Society for Neuroscience* 31 (50): 18506–21.

Connell, Anna R., Michelle B. Hookham, Dongxu Fu, Derek P. Brazil, Timothy J. Lyons, and Jeremy Y. Yu. 2022. "Comparisons of α2-Adrenergic Agents, Medetomidine and Xylazine, with Pentobarbital for Anesthesia: Important Pitfalls in Diabetic and Nondiabetic Rats." *Journal of Ocular Pharmacology and Therapeutics: The Official Journal of the Association for Ocular Pharmacology and Therapeutics* 38 (2): 156–66.

Cooper, B. G., D. Y. Miya, and S. J. Mizumori. 1998. "Superior Colliculus and Active Navigation: Role of Visual and Non-Visual Cues in Controlling Cellular Representations of Space." *Hippocampus* 8 (4): 340–72.

Dean, P. 1981. "Visual Pathways and Acuity Hooded Rats." *Behavioural Brain Research* 3 (2): 239–71.

De Franceschi, Gioia, and Samuel G. Solomon. 2018. "Visual Response Properties of Neurons in the Superficial Layers of the Superior Colliculus of Awake Mouse." *The Journal of Physiology* 596 (24): 6307–32.

Durand, Séverine, Ramakrishnan Iyer, Kenji Mizuseki, Saskia de Vries, Stefan Mihalas, and R. Clay Reid. 2016. "A Comparison of Visual Response Properties in the Lateral Geniculate Nucleus and Primary Visual Cortex of Awake and Anesthetized Mice." *The Journal of Neuroscience: The Official Journal of the Society for Neuroscience* 36 (48): 12144–56.

Foik, Andrzej T., Leo R. Scholl, Georgina A. Lean, and David C. Lyon. 2020. "Visual Response Characteristics in Lateral and Medial Subdivisions of the Rat Pulvinar." *Neuroscience* 441 (August): 117–30.

Garrity, Abigail G., Simhadri Botta, Stephanie B. Lazar, Erin Swor, Giancarlo Vanini, Helen A. Baghdoyan, and Ralph Lydic. 2015. "Dexmedetomidine-Induced Sedation Does Not Mimic the Neurobehavioral Phenotypes of Sleep in Sprague Dawley Rat." *Sleep* 38 (1): 73–84.

Gil, Rita, Ana Mafalda Valente, and Noam Shemesh. 2022. "Activation/suppression Balances in Rat Superior Colliculus Encode the Visual Continuity Illusion." *bioRxiv*. https://doi.org/10.1101/2022.11.27.518086.

Girman, S. V., Y. Sauvé, and R. D. Lund. 1999. "Receptive Field Properties of Single Neurons in Rat Primary Visual Cortex." *Journal of Neurophysiology* 82 (1): 301–11.

Grubb, Matthew S., and Ian D. Thompson. 2003. "Quantitative Characterization of Visual Response Properties in the Mouse Dorsal Lateral Geniculate Nucleus." *Journal of Neurophysiology* 90 (6): 3594–3607.

Ito, Shinya, David A. Feldheim, and Alan M. Litke. 2017. "Segregation of Visual Response Properties in the Mouse Superior Colliculus and Their Modulation during Locomotion." *The Journal of Neuroscience: The Official Journal of the Society for Neuroscience* 37 (35): 8428–43.

Katz, L. C., and C. J. Shatz. 1996. "Synaptic Activity and the Construction of Cortical Circuits." *Science* 274 (5290): 1133–38.

King, Andrew J. 2004. "The Superior Colliculus." *Current Biology: CB* 14 (9): R335–38.

Li, Xiaoyuan, Chaokui Sun, and Li Shi. 2015. "Comparison of Visual Receptive Field Properties of the Superior Colliculus and Primary Visual Cortex in Rats." *Brain Research Bulletin* 117 (August): 69–80.

Martin, P. R. 1986. "The Projection of Different Retinal Ganglion Cell Classes to the Dorsal Lateral Geniculate Nucleus in the Hooded Rat." *Experimental Brain Research. Experimentelle Hirnforschung. Experimentation Cerebrale* 62 (1): 77–88.

Nair, Govind, Moon Kim, Tsukasa Nagaoka, Darin E. Olson, Peter M. Thulé, Machelle T. Pardue, and Timothy Q. Duong. 2011. "Effects of Common Anesthetics on Eye Movement and Electroretinogram." *Documenta Ophthalmologica. Advances in Ophthalmology* 122 (3): 163–76.

Ohki, Kenichi, and R. Clay Reid. 2007. "Specificity and Randomness in the Visual Cortex." *Current Opinion in Neurobiology* 17 (4): 401–7.

Pecka, Michael, Yunyun Han, Elie Sader, and Thomas D. Mrsic-Flogel. 2014. "Experience-Dependent Specialization of Receptive Field Surround for Selective Coding of Natural Scenes." *Neuron* 84 (2): 457–69.

Piscopo, Denise M., Rana N. El-Danaf, Andrew D. Huberman, and Cristopher M. Niell. 2013. "Diverse Visual Features Encoded in Mouse Lateral Geniculate Nucleus." *The Journal of Neuroscience: The Official Journal of the Society for Neuroscience* 33 (11): 4642–56.

Prévost, François, Franco Lepore, and Jean-Paul Guillemot. 2007. "Spatio-Temporal Receptive Field Properties of Cells in the Rat Superior Colliculus." *Brain Research* 1142 (April): 80–91.

Ringach, Dario L., Patrick J. Mineault, Elaine Tring, Nicholas D. Olivas, Pablo Garcia-Junco-Clemente, and Joshua T. Trachtenberg. 2016. "Spatial Clustering of Tuning in Mouse Primary Visual Cortex." *Nature Communications* 7 (August): 12270.

Sahibzada, N., P. Dean, and P. Redgrave. 1986. “Movements Resembling Orientation or Avoidance Elicited by Electrical Stimulation of the Superior Colliculus in Rats.” *The Journal of Neuroscience: The Official Journal of the Society for Neuroscience* 6 (3): 723–33.

Scheinin, H., R. Virtanen, E. MacDonald, R. Lammintausta, and M. Scheinin. 1989. “Medetomidine--a Novel Alpha 2-Adrenoceptor Agonist: A Review of Its Pharmacodynamic Effects.” *Progress in Neuro-Psychopharmacology & Biological Psychiatry* 13 (5): 635–51.

Singh, Surender, B. G. Roy, S. G. Ramachandra, and P. Nagarajan. 2021. “Nonhuman Primates in Biomedical Research.” In *Essentials of Laboratory Animal Science: Principles and Practices*, edited by P. Nagarajan, Ramachandra Gudde, and Ramesh Srinivasan, 313–41. Singapore: Springer Singapore.

Sriram, Balaji, Philip M. Meier, and Pamela Reinagel. 2016. “Temporal and Spatial Tuning of Dorsal Lateral Geniculate Nucleus Neurons in Unanesthetized Rats.” *Journal of Neurophysiology* 115 (5): 2658–71.

Tang, Jiaying, Silvia C. Ardila Jimenez, Subhojit Chakraborty, and Simon R. Schultz. 2016. “Visual Receptive Field Properties of Neurons in the Mouse Lateral Geniculate Nucleus.” *PloS One* 11 (1): e0146017.

Tschetter, Wayne W., Gubbi Govindaiah, Ian M. Etherington, William Guido, and Cristopher M. Niell. 2018. “Refinement of Spatial Receptive Fields in the Developing Mouse Lateral Geniculate Nucleus Is Coordinated with Excitatory and Inhibitory Remodeling.” *The Journal of Neuroscience: The Official Journal of the Society for Neuroscience* 38 (19): 4531–42.

Tsurugizawa, Tomokazu, Yukari Takahashi, and Fusao Kato. 2016. “Distinct Effects of Isoflurane on Basal BOLD Signals in Tissue/vascular Microstructures in Rats.” *Scientific Reports* 6 (December): 38977.

Vreysen, Samme, Bin Zhang, Yuzo M. Chino, Lutgarde Arckens, and Gert Van den Bergh. 2012. “Dynamics of Spatial Frequency Tuning in Mouse Visual Cortex.” *Journal of Neurophysiology* 107 (11): 2937–49.

Wang, Lupeng, Rashmi Sarnaik, Krsna Rangarajan, Xiaorong Liu, and Jianhua Cang. 2010. “Visual Receptive Field Properties of Neurons in the Superficial Superior Colliculus of the Mouse.” *The Journal of Neuroscience: The Official Journal of the Society for Neuroscience* 30 (49): 16573–84.

Xu-hong, Z. U. O., S. H. I. Xue-feng, X. I. E. Fang, X. U. Li-min, Zhao Kan-xing, and Others. 2012. “Development of Spatiotemporal Frequency Turning in Rat Lateral Geniculate Neuron.” *Chinese Journal of Experimental Ophthalmology*, 388–91.

Yildirim, Funda, Joana Carvalho, and Frans W. Cornelissen. 2018. “A Second-Order Orientation-Contrast Stimulus for Population-Receptive-Field-Based Retinotopic Mapping.” *NeuroImage* 164 (January): 183–93.

Zhang, Xian, Xu An, Hanxiao Liu, Jing Peng, Shanshan Cai, Wei Wang, Da-Ting Lin, and Yupeng Yang. 2015. “The Topographical Arrangement of Cutoff Spatial Frequencies across Lower and Upper Visual Fields in Mouse V1.” *Scientific Reports* 5 (January): 7734.
